# Patient‐derived organoids in cellulosic sponge model chemotherapy response of metastatic colorectal cancer

**DOI:** 10.1002/ctm2.285

**Published:** 2021-01-12

**Authors:** Yanjie Xu, Jianjun Chen, Yizhou Huang, Yang Luo, An‐Chih Hsieh, Jianyi Chen, Han Li, Xunbin Wei, Wei‐Qiang Gao, Ming Zhong, Yan Zhang

**Affiliations:** ^1^ Med‐X Research Institute & School of Biomedical Engineering Shanghai Jiaotong University Shanghai China; ^2^ State Key Laboratory of Oncogenes and Related Genes Renji‐Med X Stem Cell Research Center, Renji Hospital, School of Medicine Shanghai Jiaotong University Shanghai China; ^3^ Department of Gastrointestinal Surgery, Renji Hospital, School of Medicine Shanghai Jiaotong University Shanghai China; ^4^ Pishon Biomedical Co., Ltd Taipei Taiwan China; ^5^ Biomedical Engineering Department Peking University Beijing China; ^6^ Key Laboratory of Carcinogenesis and Translational Research (Ministry of Education/Beijing) Peking University Cancer Hospital & Institute Beijing China

Dear Editor,

Patient‐derived organoids (PDOs) closely recapitulate human colorectal cancer biology and have recently emerged as preclinical models for personalized therapy design.^1^ However, PDOs cultured in Matrigel (PDOs^Matrigel^) failed to predict outcome for treatment with the first‐line FO chemotherapeutic regimen (5‐fluorouracil plus oxaliplatin) for metastatic colorectal cancer (mCRC) patients.^2^ In this study, we establish in vitro culture conditions of mCRC‐PDOs in a hydroxypropyl cellulose allyl conjugated with collagen (HA‐Coll sponge),^3^ and utilized this system to examine the drug sensitivity of FO regimen (Figure 1A).

**FIGURE 1:**
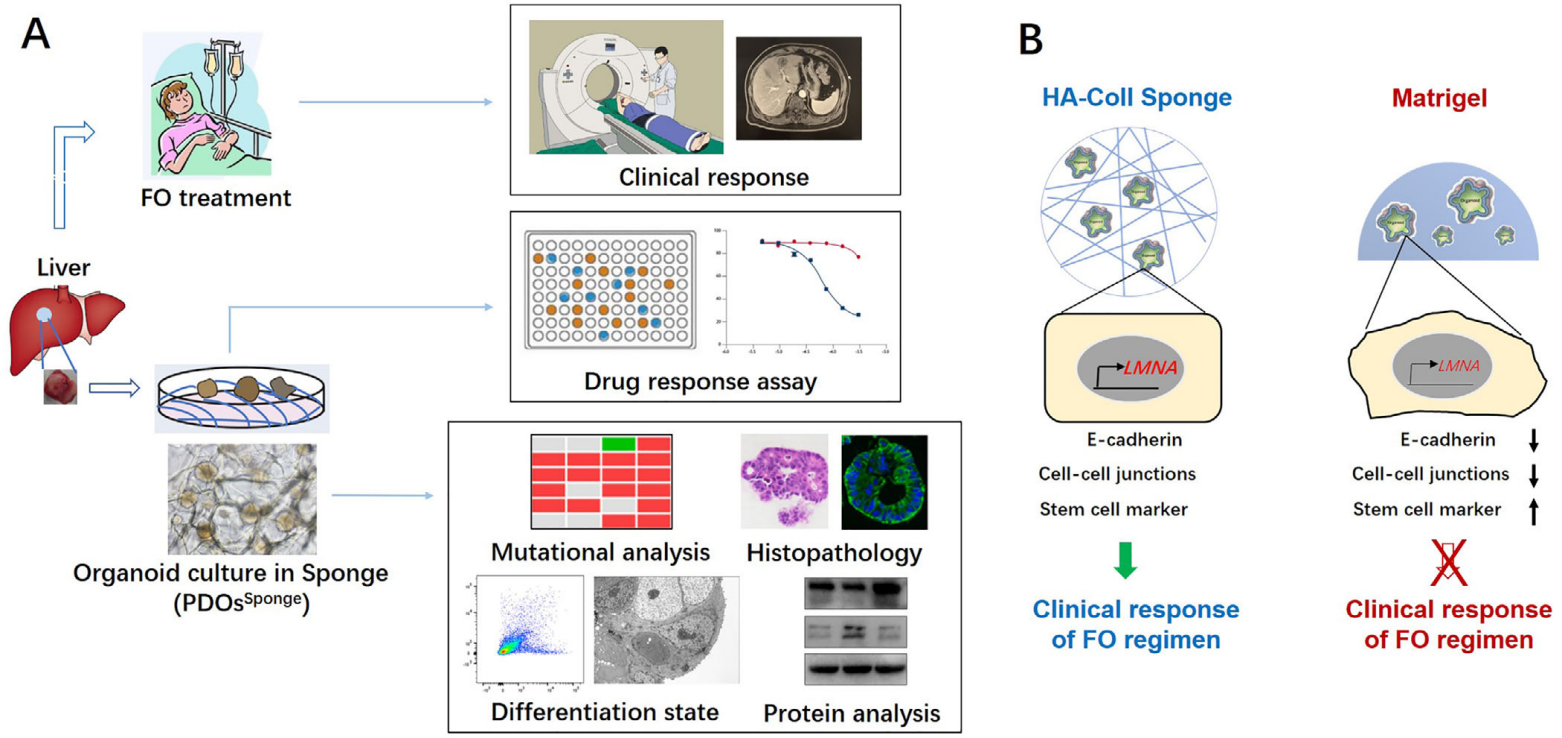
The experimental design and graphic mechanism. (A) Image‐guided biopsies were used to generate PDOs from liver metastatic colorectal cancers. PDOs were established from biopsies obtained before FO treatment in HA‐Coll sponge. Histopathology, molecular profiling, differentiation state, and protein analysis of PDOs and their parental tissues were characterized and compared. PDOs were used for FO drug screening, and ex vivo response to FO regimen was compared with clinical response. (B) The physical elasticity (E) of HA‐Coll sponge is close to that of the colorectal tissue, so that PDOs in HA‐Coll sponge could keep the similar expression level of lamin‐A as their parental tumor tissues. The lamin‐A protein could modulate the differentiation of colorectal epithelial cell to keep their epithelial state, which is important for their drug‐sensitivity on FO chemotherapeutic regimen. However, PDOs in Matrigel have lower expression level of lamin‐A and acquire mesenchymal characteristics, thereby they gradually form the drug resistance to FO regimen

A total of 12 tumor organoid cultures from metastatic lesion were developed from 12 patients enrolled in Renji hospital between September 2018 and October 2019 (Table S1). In the HA‐Coll sponge, the primary tumor cells organized into 3D spheroids within 3 days, although its size was smaller than that of organoid grown in Matrigel, and continues to grow until reaching similar sizes at about day 7 and its size was relative homogeneous (Figure 2A, Figure S1A and B). PDOs in HA‐Coll sponge (PDOs^Sponge^) proliferated at the same level as PDOs^Matrigel^ in the next generation (Figure 2B and C, Figure S1C and D). H&E staining showed notable morphological similarity among PDOs^Sponge^ and parental patient biopsies, and the “cystic versus solid” structure of the epithelium were preserved (Figure 2D, Figure S1E); the typical expression pattern of CDX‐2 (caudal type homeox‐2) and CK7 (keratin 7) in CRC was observed in PDOs^Sponge^ (Figure S1F). Collectively, the mCRC‐PDOs were successfully cultured in HA‐Coll sponge.

**FIGURE 2:**
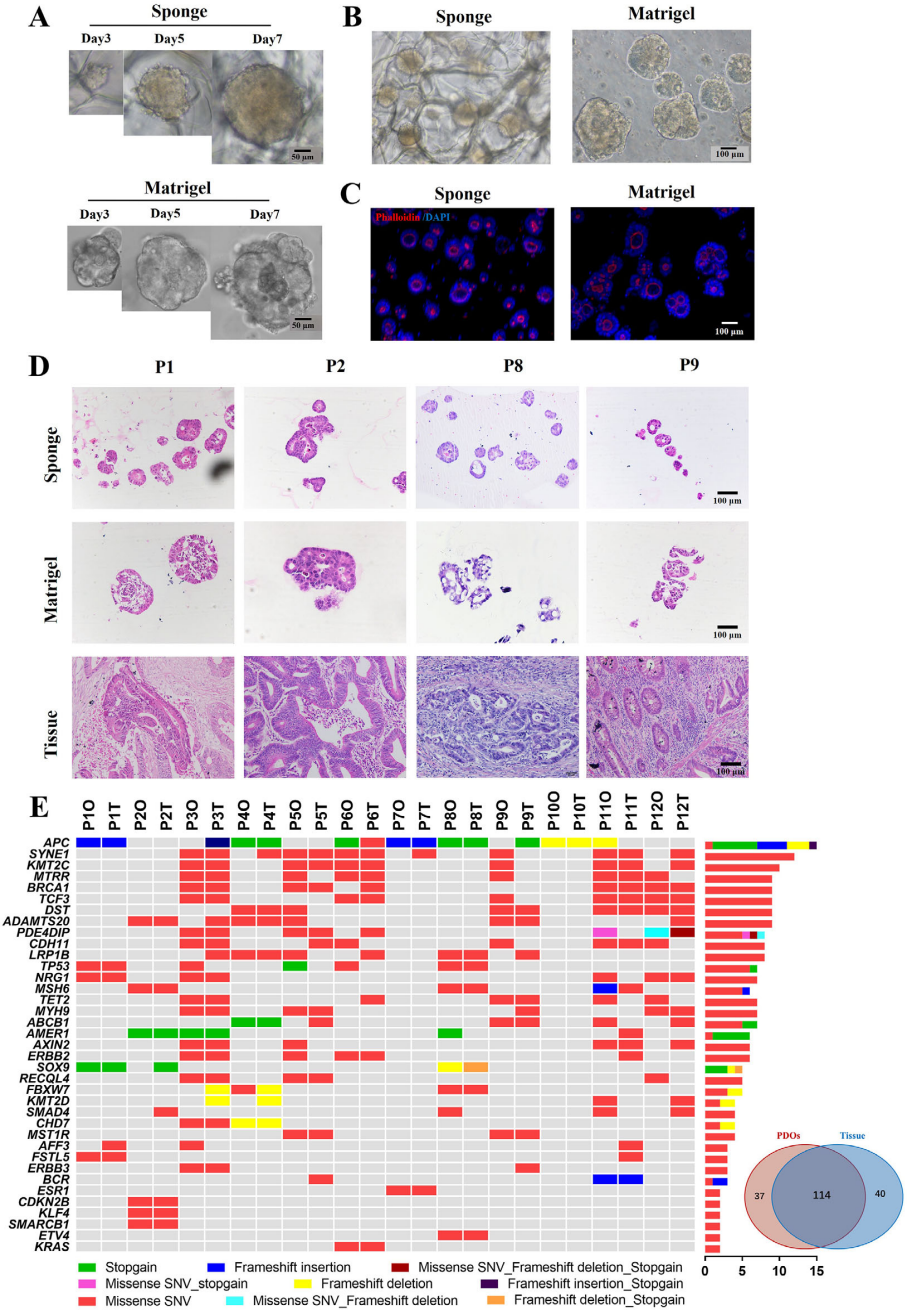
Phenotype and genotype of mCRC patient‐derived organoids (mCRC‐PDOs) in HA‐Coll sponges. Freshed isolated tumor cells derived from mCRC patients were seeded into HA‐Coll sponges or Matrigel in 48‐well plates (5 × 10^3^/well). (A) Representative time course of the mCRC organoids from the same parental biopsy in HA‐Coll sponges (top) or Matrigel (bottom). (B) Representative phase‐contrast images of mCRC‐PDOs derived from the same parental biopsy in HA‐Coll sponges or Matrigel 14 days after implantation. (C) Representative 3D confocal fluorescence images of mCRC‐PDOs derived from the same parental biopsy in HA‐Coll sponges or Matrigel 14 days after implantation. Phalloidin for cytoskeleton(red). Counterstain is DAPI (blue). (D) PDOs^Sponge^ reconstitute the histology and morphology of parental tissue. H&E staining of PDOs from FO‐sensitive PR patients comparing to their parental tumors. (E) Molecular characterization of mCRC‐PDOs in HA‐Coll sponges. Heatmap showing the most frequently mutated genes in PDOs^Sponge^ and their parental tissues. Venn diagram demonstrating 74% mutational overlap between PDOs^Sponge^ and parental tissue biopsies

To explore whether PDOs^Sponge^ keep the genetic characteristics of the parental tumor, the next‐generation sequencing (NGS) was used to examine 561 cancer‐related genes in 12 pairs of PDOs^Sponge^ and their parental tumor tissues. The molecular landscape of the PDOs^Sponge^ largely overlapped with parental tumor and a 74% overlap in mutational spectrum was observed between these two groups (Figure 2E, Table S2), indicating that the major mCRC molecular genotypes are represented in PDOs^Sponge^.

Next, we examined the predictive value of mCRC‐PDOs^Sponge^ on FO regime by comparisons of their ex vivo response data with clinical responses in patients. Twelve patients enrolled in our study were applied with FO therapy in clinic and the PDOs^Sponge^ were established from the biopsy before the treatment (Table S1, Figure 3A and Figure S2). The PDOs^Sponge^ were treated with different concentrations of 5‐FU and oxaliplatin, and after 6 days the growth rates were examined by Cell‐Titer Glo2.0 assay. We fitted dose–response curves (DRCs) and quantified responses to FO by calculating the IC50 and the area under the DRC (AUC_DRC_), both of which were significantly different between PDOs^Sponge^ from PR/SD versus PD lesions (Figure 3B–D). However, there were no significant difference on IC50 or AUC_DRC_ in the PDOs cultured in Matrigel (Figure 3E–G). In addition to FO regimen, irinotecan‐based chemotherapy is also used for CRC patients as second‐line regimen.^4^ Figure S3 showed that there were no significant differences on IC50 between PDOs^Sponge^ and PDOs^Matrigel^, suggesting that the distinction of these two groups mainly reflect the clinical response to FO chemotherapy but not irinotecan‐based regimen and highlighted the clinical potential of PDOs^Sponge^ for FO therapeutic selection in mCRC.

**FIGURE 3:**
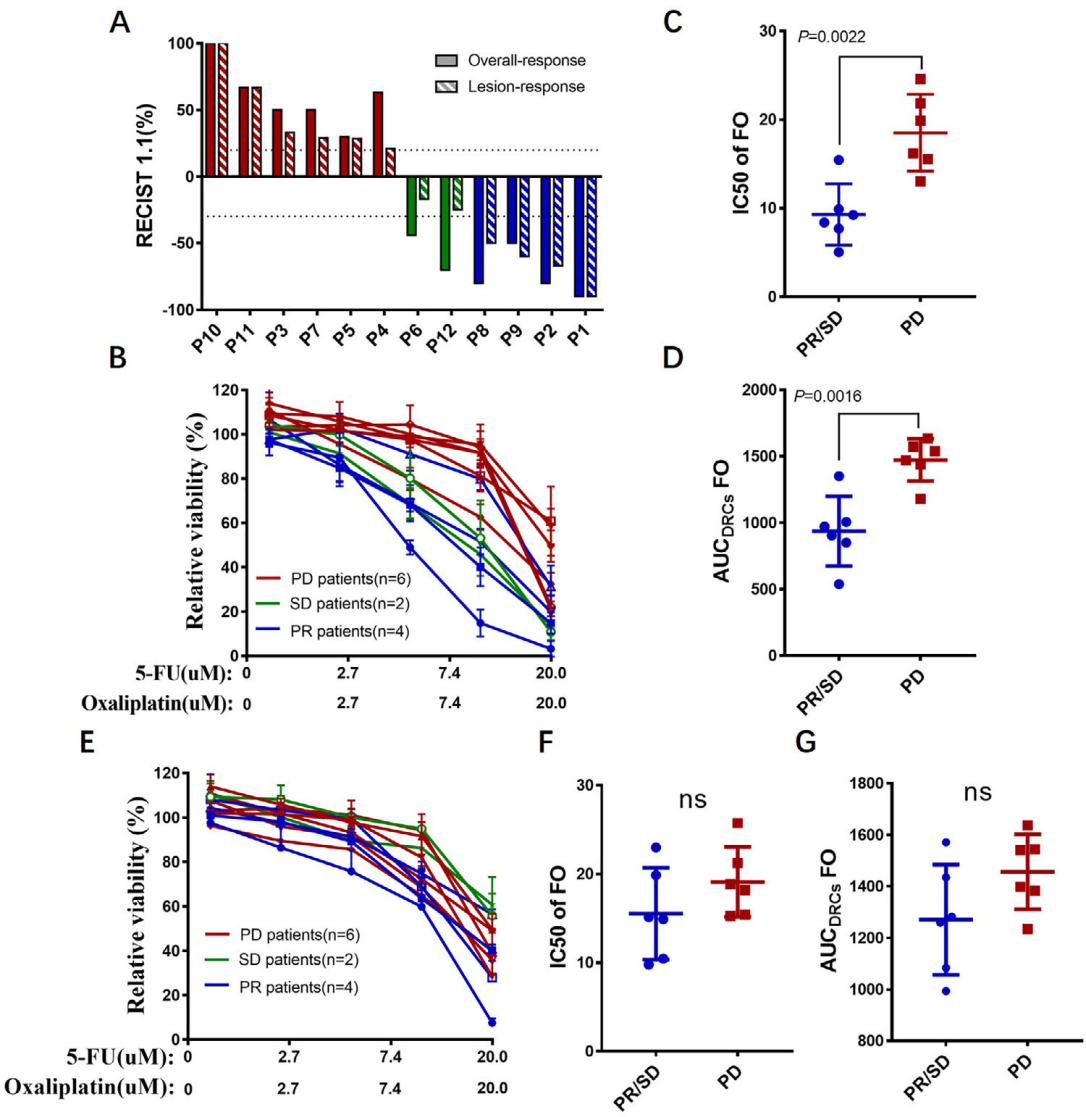
PDOs in HA‐Coll sponges can predict drug response of metastatic colorectal patients to FO therapy. (A) Waterfall plot of 12 patients’ overall responses and best responses of the biopsied lesion in the FO‐treated PDOs cohort. Red indicates PD, green indicates SD, and blue indicates PR (PR, partial response; SD, stable disease; PD, progressive disease). (B) Fitted dose‐response curves (DRCs) of 12 PDOs^Sponge^ exposed to FO in vitro. Red lines represent PDOs from PD patients(*n* = 6), green lines represent PDOs derived from SD patients (*n* = 2), and blue lines represent PDOs derived from PR patients (*n* = 4). IC50 represent in vitro sensitivity of PDOs to FO. (C and D) The IC50 values were quantified and the area under the DRC (AUC_DRC_) was calculated by integrating the DRC of each PDO in (B). (E–G) Drug sensitivity of PDOs in Matrigel does not predict clinical response of parental colorectal patients to FO therapy. (E) Fitted dose–response curves (DRCs) of 12 PDOs^Matrigel^ exposed to FO in vitro. (F and G) The IC50 values were quantified and the area under the DRC (AUC_DRC_) was calculated by integrating the DRC of each PDO in (E)

Epithelial to mesenchymal transition (EMT) has been demonstrated to play important roles in therapeutic resistance,^5^ so we wondered if EMT was involved in the differential drug‐sensitivity between PDOs^Sponge^ and PDOs^Matrigel^. First, the expression of E‐cadherin in PDOs and the parental tumors from PR patients was examined by immunofluorescence and the results showed that PDOs^Sponge^ had similar expression level of E‐cadherin as their correspondent parental tissues, while it was dramatically decreased in PDOs^Matrigel^ (Figure 4A, Figure S4A). These results were confirmed by the data of western blotting (Figure S5A‐H) and by the qPCR results of other EMT‐related makers (Figure S5I). In addition, activation of the EMT usually elicits changes in cell morphology^6^ and the results of the transmission electron microscopy (TEM) demonstrated that cell–cell junctions were observed in both PDOs^Sponge^ and parental biopsies, while the intercellular connections in the PDOs^Matrigel^ were much looser and minimal (Figure 4B, Figure S4B). Next, we utilized EMT inhibitors, methacycline HCl or (E)‐SIS3, to inhibit the progression of EMT (Figure S6A–C) and the ex vivo drug‐sensitivity experiments showed that, after adding methacycline HCl, the IC50 values of FO in PDOs^Matrigel^ were significantly reduced (Figure 4C, Figure S4C). The same tendency was also observed in those treated with (E)‐SIS3 (Figure 4D, Figure S4D), suggesting that blocking EMT in PDOs^Matrigel^ could recover its drug‐sensitivity in FO chemotherapy; however, this recovery was not observed in irinotecan‐treated groups (Figure S6D and E). Whether EMT inhibitors could be used in PDO culture for drug screening will need more experiments to confirm, particularly in different chemotherapeutic drug combination. Conclusively, our results indicated that PDOs^Sponge^ remained the original epithelial state of parental tumor tissues, which is important for the drug‐sensitivity of FO, but not for irinotecan.

**FIGURE 4:**
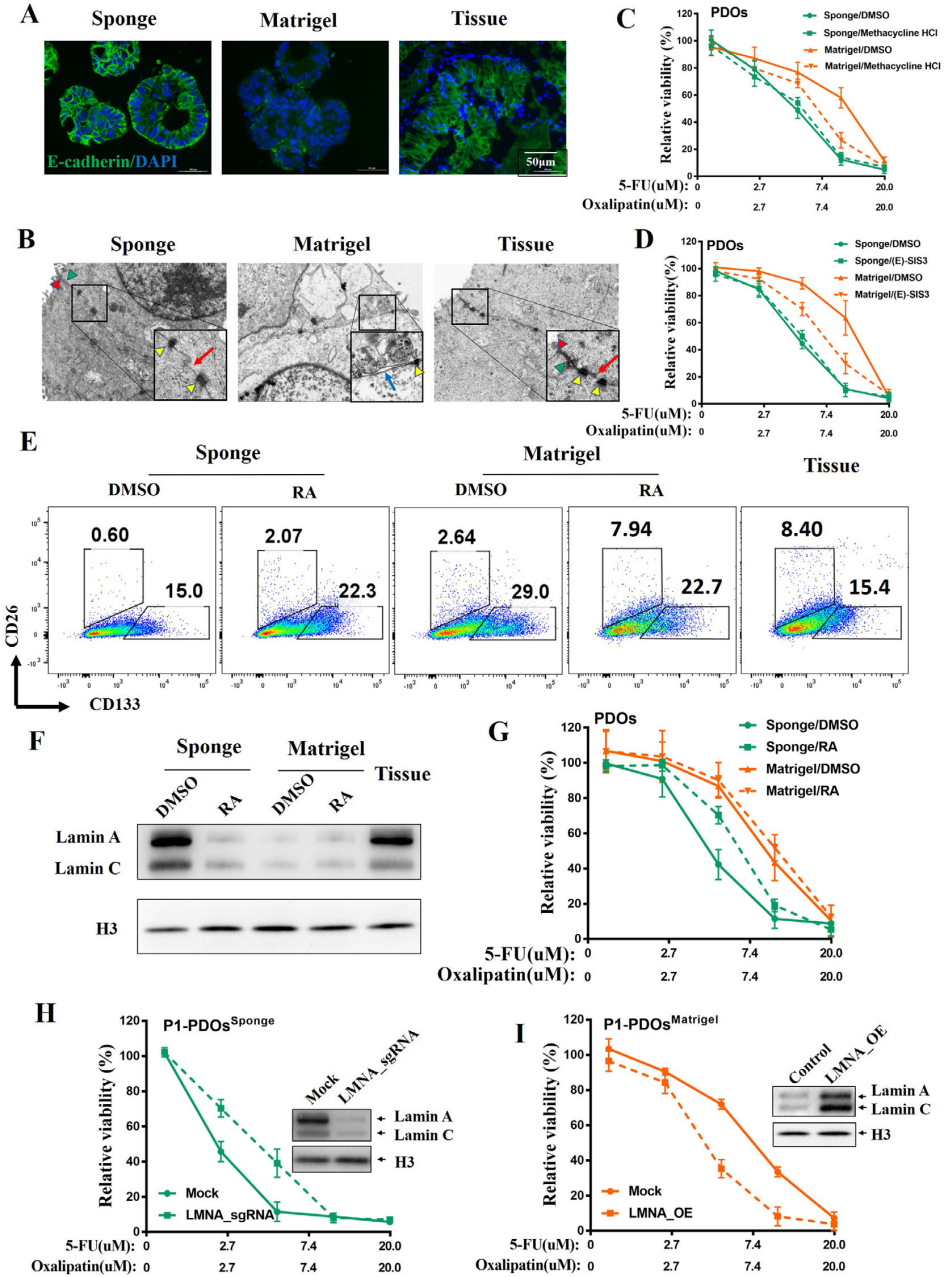
HA‐Coll sponge can keep the epithelial and differentiation state of PDOs. (A) The expression levels of epithelial marker E‐cadherin of representative P1 were examined by immunofluorescence. The epithelial cells of parental biopsy were isolated as control. (B) Cell–cell junctions in PDOs^Sponge^ and PDOs^Matrigel^ from representative P1 were examined by transmission electron microscopy. Red arrowhead for tight junctions; Green arrowhead for adherens junctions; yellow arrowhead for desmosome; red arrow for gap junctions; blue arrow for dissolution of cell–cell junctions. (C and D) The ex vivo dose‐response curves (DRCs) of representative PDOs from P1 exposed to FO regimen after adding the EMT inhibitors: Methacycline HCl (10 µM) or (E)‐SIS3 (3 µM). (E) Lamin‐A contribute to differentiation state of PDOs^Sponge^. The proportion of CRC‐CSCs in PDOs and parental tissues from P1 was analyzed by flow cytometry. PDOs were pretreated with 5‐FU and oxaliplatin for 6 days with or without 1 µM retinoic acid (RA). (F) The expression levels of lamin A protein in PDOs and their parental tumor tissues from P1 were examined by immunoblotting. (G) The DRCs of PDOs from P1 treated with 5‐FU and oxaliplatin in the presence of RA. (H) The DRCs of PDOs^Sponge^ from P1 treated with 5‐FU and oxaliplatin after *LMNA* KO. (I) The DRCs of PDOs^Matrigel^ from P1 treated with 5‐FU and oxaliplatin after *LMNA* overexpressed

The epithelial state of cells usually reflected their differential states,^7^ so we used CD133 and CD26 antibodies to examine the percentage of CSCs in the PDOs by flow cytometry. Figure 4E and Figure S4E demonstrated that the percentage of CD133^+^ cells in PDOs^Matrigel^ was obviously higher than those in PDOs^Sponge^ and parental tumor tissue, although CD26 maybe was not proper marker for CSCs in our experiments. Mechanical tension on a cell from its environment control gene expression to modulate tissue‐specific differentiation^8^ and stiff environment could increase the transcription of lamin‐A to alter nuclear rheology, thereby promoting cell differentiation.^9^

To figure out if lamin‐A was involved in keeping the epithelial state in PDOs^Sponge^, the expression level of lamin‐A in nuclear proteins was examined by western blotting. Figure 4F and Figure S4F showed that the expression of lamin‐A was dramatically decreased in PDOs^Matrigel^, but not in PDOs^Sponge^, which was consistent with the previous studies that the matrix stiffness of Sponge^3^ is at the similar level to that of intestinal epithelial cells.^10^ Next we added retinoic acid (RA) to inhibit the expression level of lamin‐A in PDOs^Sponge^ (Figure 4F, Figure S4F),^9^ and thereby enhance the percentage of CSCs in PDOs^Sponge^ (Figure 4E, Figure S4E), consequently, the IC50 value of FO in PDOs^Sponge^ was dramatically increased by RA (Figure 4G, Figure S4G). To further confirm these results, we knocked out *LMNA* in PDOs^Sponge^ and overexpressed *LMNA* in PDOs^Matrigel^ to perform the drug response assay. The same tendency with RA results was observed (Figure 4H and I, Figure S7), suggesting that lamin‐A contributes to the epithelial state of PDOs^Sponge^.

In summary, we reported that the mCRC‐PDOs^Sponge^ could recapitulate the phenotype and genotype of the parental tumor tissue. PDOs^Sponge^ could keep the similar expression level of lamin‐A as their parental tumor tissues, maintain the epithelial state of colorectal epithelial cell, and predict drug responses to FO chemotherapeutic regimen (Figure 1B).

## CONFLICT OF INTEREST

The authors declare that they have no conflict of interest.

## AUTHOR CONTRIBUTIONS

Yan Zhang and Yanjie Xu: Conceptualization, methodology, and writing and original draft preparation. Jianjun Chen and Ming Zhong: Funding acquisition and data curation. Yizhou Huang and Yang Luo: Resources, visualization, and investigation. An‐Chih Hsieh and Jianyi Chen: Resources. Han Li: Software and validation. Xunbin Wei, Wei‐Qiang Gao, and Ming Zhong: Project administration and supervision. Yanjie Xu and Yan Zhang: Writing, reviewing, and editing.

## Supporting information

Supporting Information

Supporting Information

Supporting Information
