# Supplementary material for: Patient‐derived organoids in cellulosic sponge model chemotherapy response of metastatic colorectal cancer
Source: Clin Transl Med. 2021 Jan 12;11(1):e285. doi: 10.1002/ctm2.285 (PMC7803352; doi:10.1002/ctm2.285)
Supplement: Supplementary file 1 — Supporting Information [file CTM2-11-e285-s001.docx]

Supporting Information

**Patient-derived organoids in cellulosic sponge model chemotherapy response of metastatic colorectal cancer**

Yanjie Xu, Jianjun Chen, Yizhou Huang, Yang Luo, An-Chih Hsieh, Jianyi Chen, Han Li， Xunbin Wei*, Wei-Qiang Gao*, Ming Zhong*, and Yan Zhang*

**Methods**

**Study Design**

The objectives of the research were to establish and characterize the [alternative](javascript:;) HA-Coll sponge-based mCRC-PDOs culture platform, and investigate their subsequent drug-screening potentials to recapitulate *ex vivo* responses to chemotherapeutic agents observed in the clinic. Biopsies obtained from mCRC patients were used to establish a biobank of PDOs^Sponge^. Written informed consent was provided from all patients. Some representative PDOs were established for direct comparison between clinical and *in vitro* drug-screening response. Responses of the biopsied lesion of clinical patients were scored using RECIST 1.1. The histopathological and genomic profiling of PDOs^Sponge^ were characterized in comparison to their parental tumor.

To explore if PDOs^Sponge^ can recapitulate patient response to FO, we examined the predictive value of PDOs^Sponge^ by comparisons of patient clinical responses with *ex vivo* responses to FO. To explore if EMT is involved in the differential drug-sensitivity of PDOs, we examined the expression level of EMT related markers in PDOs^Sponge^, PDOs^Matrigel^ and parental tissue by immunofluorescence and western blotting. To analyze the changes in cell morphology and physiology, we observed cell-cell junctions of parental tissues and PDOs^Sponge^ or PDOs^Matrigel^ by transmission electron microscopy. To explore the essential role of epithelial state of PDOs on the drug-sensitivity of FO, we utilized EMT inhibitors to block the progression of EMT and observed their effects on the growth rates of PDOs treated with FO. To figure out if lamin-A was involved in keeping the epithelial state in PDOs^Sponge^, the expression of lamin-A- was examined by western blotting. We also added RA, the inhibitor of lamin-A expression, to observe its effects on drug-sensitivity of FO.

**Human Tissues**

All samples used for organoid establishment and biological analyses were obtained from Renji Hospital with written informed consent. The study was approved by the ethical committee of Renji Hospital affiliated Shanghai Jiao Tong University. Neoplastic tissues were obtained from biopsy specimens. we avoided samples with low cellularity or contamination by obtaining core and clean biopsies, together with direct assessment by a pathologist to evaluate the composition (ie, to ensure its normal/tumor status) using histological methods. All patients were diagnosed with mCRC. Patients were treated according to clinically approved FO regimen. The information of CRC patients was obtained by review of electronic medical records. Prognostic information was acquired by follow-up.

**Establishment and culture of PDOs in HA-Coll sponges**

Biopsies from mCRC patients were collected in cold PBS for a maximum of 24 hours at 4°C before being minced, and subsequently digested in 5ml enzyme buffer for 60 minutes at 37℃ while shaking. The composition of digestion enzyme buffer was Collagenase type Ⅳ(2mg/ml), Hyaluronidase (200μg/ml), DNaseⅠ(200μg/ml) and Y-27632 (10μM). For thoroughly digestion, the remaining fragments were additionally dissociated with TrypLE Express (Invitrogen) at 37℃ for additional 15 min. Tissue fragments were discarded through 70μm Nylon Cell Strainer and the dissociated cells were collected, centrifuged (400g ,5min, 4℃), resuspended in culture medium and 5x10^3^ cells/25μl were dispensed into HA-Coll sponges (produced and supplied by PISHON Biomedical, Taiwan) which were pre-plated at 48-well flat bottom cell culture plates (Corning). After 4 hours’ incubation in a 37℃ and 5% CO2 cell culture incubator, extra 250μl culture medium was added into wells to submerge sponges. PDOs in Matrigel were cultured as previously description.^1^

PDOs in sponges can be passaged using TrypLE Express (Invitrogen). Briefly, sponges were mechanically torn by [tweezers](javascript:;) to release the tumor organoids and organoids were collected in a tube and centrifuged at 400g for 5min at 4℃ to remove the medium. 1ml TrypLE Express containing 1 mM EDTA was added and PDOs were incubated at 37℃ for 5-10min, after then, PDOs^Sponge^ were harvested out by mechanically pipetting using 200μl yellow pellet tips. The isolated PDOs^Sponge^ fragments were then washed with PBS, pelleted, resuspended in culture medium and re-seeded at an appropriate ratio of 1:2 to 1:5. PDOs^Sponge^ can be biobanked using FBS containing 10% DMSO and cryopreserved in liquid nitrogen.

The isolation of tumor epithelium cells and Matrigel-based organoid culture was performed as previous description.^1^ For the regular organoid culture, the percentage of Matrigel (Corning, Cat#356231) was almost over 90% (medium with cells: Matrigel=1:10).

**Human PDO culture media**

| Additive | Supplier | Cat.No. | Concentration |
| --- | --- | --- | --- |
| Advanced DMEM/F12 | Gibco | 12634010 | 1X |
| EGF | PeproTech | AF-100-15 | 50ng/ml |
| Noggin | PeproTech | 96-120-10C | 100ng/ml |
| R-Spondin 1 | PeproTech | 96-120-38 | 500ng/ml |
| Gastrin | Sigma-Aldrich | G9145 | 10nM |
| Y-27632 | Sigma-Aldrich | Y0503 | 10μM |
| Nicotinamide | Sigma-Aldrich | N0636 | 10mM |
| N-acetylcysteine | Sigma-Aldrich | A9165-5G | 1mM |
| A83-01 | Sigma-Aldrich | SML0788 | 500nM |
| SB202190 | Sigma-Aldrich | S7067 | 10μM |
| GlutaMAX-I | Gibco | G9145 | 1X |
| HEPES | Gibco | 15630106 | 1X |
| B27 | Gibco | 17504044 | 1X |
| N2 | Gibco | 17502048 | 1X |

**Histology and Immunostaining Procedures**

Structure maintained PDOs in sponges were harvested out by [mechanical](javascript:;)ly tearing apart sponges and PDOs in Matrigel were harvested out using Cell Recovery solution (Corning). And Then fixed in 10% formalin for 1h after washed in ice-cold PBS and pelleted(400g,5min,4℃). The pellet was placed in 50µl rat tail collagen I (Corning) and incubated at 37℃ for 30min allowing the collagen pellet to solidify before processing and embedding. Tissue and organoids were embedded in 200 µl OCT compound, frozen and stored at -80℃. Or embedded in paraffin. Sections were subjected to H&E as well as immunohistochemical staining. H&E staining and immunohistochemistry staining for CDX-2 and CK7 of PDOs and tissues were conducted following a standard staining protocol on 5 μm paraffin sections.

For immunostaining, 5 μm freezing organoids and tissue sections were washed in PBS, ﬁxed with 4% paraformaldehyde, permeabilized with 0.3% Triton X-100,and then blocked in 10% donkey serum for 1h at room temperature, followed by incubation with primary antibodies overnight at 4°C. Samples were then incubated with secondary antibodies labeled with Alexa Fluor 488(Invitrogen) at RT for 1 h. Slides were mounted with VECTASHIELD mounting medium with DAPI(Invitrogen). Immunofluorescence staining was imaged using a Leica spectral confocal microscope.

**DNA sequencing**

For targeted library preparation and DNA-sequencing, DNA samples from 12 PDOs and PDO-matching tumors were extracted using the AllPrep DNA/RNA/miRNA universal kit (Qiagen) according to manufacturer’s protocol. The quality of DNA samples was confirmed by agarose gel electrophoresis for the degree of DNA degradation and the presence of miscellaneous bands, RNA and protein contamination. The DNA (≥0.5 µg) was fragmented for target resequencing. Targeted library preparation and DNA-sequencing were outsourced to Novogene (China). In brief, paired-end libraries were prepared with a Sure Select XT Custom kit (Agilent) that targets 561 cancer-related genes, and sequenced by Illumina HiSeq PE150 (Pair End 150 bp). The generated fastq files were mapped onto human reference genome version (human_B37) using Burrows-Wheeler Aligner (version 0.1.22) and Samblaster. Duplicated reads were removed using Sambamba (version 0.4.7). Somatic mutations, SNV, and Somatic INDEL were detected using muTect (version 1.1.4) and Strelka (version 1.0.13). Reference normal tissue DNA samples were available for these four patients, and variants were additionally filtered using normal DNA for these PDOs and primary tumors.

**Western blotting**

Western blot analysis protein lysates were harvested from the PDOs and primary tumor epithelium isolation using 1×RIPA lysis buffer (Thermo Scientific, USA) containing 1:100 protease inhibitor cocktail (Sigma-Aldrich) and 1:100 phosphatase inhibitor cocktail 2 (Sigma-Aldrich) and kept on ice for 30 min before being centrifuged at 10,000g for10 min at 4 °C to pellet the cell debris and obtain the supernatant. For primary tumor epithelium isolation,we purified epithelial cells by FACS from parental fresh CRC samples by epithelial tumor cells (EPCAM+), leukocytes (CD45-), endothelial cells (CD31-), and fibroblasts (FAP-).^2^ Nuclei protein lysates were harvested by Nuclear protein and cytoplasmic protein extraction kit (Beyotine). The protein lysates were electrophoresed in a 10% SDS-PAGE gel and transferred to polyvinylidene ﬂuoride membranes (Millipore). Primary antibodies were diluted in TBST containing 1% BSA, and staining was conducted overnight at 4℃ after blocking using 5% skimmed milk at room temperature. The primary antibodies used are as follows: E-cadherin (1:1000, Cell Signaling Technology),vimentin (1:1000, Cell Signaling Technology) and β-Actin (1:2000, Cell Signaling Technology); Lamin A/C (1:1000, Cell Signaling Technology) and H3(1:1000, Cell Signaling Technology). The protein bands were detected using the corresponding HRP-conjugated secondary antibody (1: 5000, Cell Signaling) for 1h at room temperature. Antibodies were diluted in TBST containing 1% BSA. Finally, signals were visualized with an enhanced chemiluminescence kit (Millipore) and then analyzed using ImageJ software (NIT, Bethesda, Maryland).

**3D PDO Drug Screening**

Drug screening of PDOs^Matrigel^ was performed according to previous studies,^3^ organoids cultured in 90%Matrigel of passage 2 or 3 were collected 4-5 days after passaging and resuspended in 2% Matrigel/organoid culture medium (15-20,000 organoids/ml) and dispensed into ultralow-attachment 96-well plates in triplicate.

For PDOs^Sponge^, PDOs in HA-Coll sponges of passage 2 or 3 were harvested 4-5 days after passaging, digested, mechanically dissociated by pipetting, resuspended culture media (15-20,000 organoids/ml) and dispended into 96-well plates prior lied with sponges in triplicate. Then, the drug adding procedure was all the same as PDOs^Matrigel^. Differential concentrations of the drug combinations were added and drug-containing medium was replaced every 2 days. And after 6 days incubation, media were removed and replaced with CellTiter-Glo 3D (Promega)-containing media according to the manufacturer’s instructions. After two hours, ATP levels were examined according to the manufacturer’s instructions.

**Lentiviral Transduction of Organoids**

PDOs^Matrigel^ were transferred to a 15-ml Falcon tube and dissociated with a glass pipette and pipetted about ten times up and down. Organoid fragments were incubated with TripLE Express (Invitrogen) for 5 min at 37 °C to broke down the fragments to small cell clusters. Medium containing 5% serum was added and cells were spun down at 1,000g for 5 min, discarded supernatant and resuspended cell clusters in a small volume of infection medium. Cell clusters were combined with 250μl SV40-LMNA-puromycin viral suspension and transferred into a 48-well culture plate. The plate was centrifuged at 600g at 32 °C for 60 min (spinoculation). The plate was placed for another 6 h in an incubator at 37 °C. Then, we collected cells, transferred them into 1.5 ml Eppendorf tube and spun them down 1,000g for 5 min. Then we discarded the supernatant, resuspended the pellet in100ul Matrigel (BD Biosciences) and split it into two wells of a 24-well culture plate. We added 500 μl of infection medium without polybrene. Two days after infection we changed medium to growth medium plus puromycin (2μg/ml). Cells were selected for 72 hr followed by a further 48 hr.

**CRISPR/Cas9 mediated *LMNA* knockout in Organoids**

Knockout organoids were generated using CRISPR/Cas9 techniques according to the previous study^4^. Single guide RNAs (sgRNAs) were designed using CRISPR (https://crispor.tefor.net/) and constructed into lentiCRISPRv2 vector (Addgene plasmid # 52961). LMNA_sgRNA: Top: 5’-CACCGCATCGACCGTGTGCGCTCGC-3’, bottom: 5’-AAACGCGAGCGCACACGGTCGATGC-3’. The organoid lipofection protocol is described in detail^5^. Human organoids were grown in expansion media, and trypsinized for 10 min at 37 °C by TrypLE Express. After trypsinization cells were resuspended in 450µl growth medium (expansion media plus Y-27632), and plated in 48 well plates at high density (80-90% confluent). Nucleic acid Lipofectamine® 2000 complexes were prepared according to the standard Lipofectamine® 2000 protocol. 4µl of Lipofectamine® 2000 reagent in 50µl Opti-MEM® medium, and a total of 1.5 µg of DNA (lentiCRISPRv2-sgRNA-puro in 50µl Opti-MEM® medium) were mixed together, incubated for 5 min, and added to the cells (50µl per well). The plate was centrifuged at 600g at 32 °C for 1 h, and incubated for 4 h at 37°C before single cells were plated in Sponge. Growth medium plus Y-27632 was exchanged with selection medium 3 days after transfection and continue 2 weeks.

**Quantitative RT-PCR**

Organoids or tumor tissues were homogenized with 1mL TRI reagent to extract total RNA. cDNA was synthesized by reverse transcription of total RNA. The expression levels of target gene were determined using TaqMan Universal Master Mix II and GAPDH TaqMan probe was used as an internal control. The primers used in each reaction were as follows: E-cad, forward 5’-ACAGCCCCGCCTTATGATTCTC-3′ and reverse 5’-AAGCGATTGCCCCATTCGTT-3′; Snail, forward 5’-CGCGCTCTTTCCTCGTCAG-3′ and reverse 5’-TCCCAGATGAGCATTGGCAG-3′; Slug, forward 5′-TTCGGACCCACACATTACCT-3′ and reverse 5′-GCAGTGAGGGCAAGAAAAAG-3′; ZEB1, forward 5′-TACAGAACCCAACTTGAACGTCACA-3′ and reverse 5′- GATTACACCCAGACTGCGTCACA-3′; and GAPDH(as internal control), forward 5′- TGACTTCAACAGCGACACCCA-3′and reverse 5′-CACCCTGTTGCTGTAGCCAAA−3′. The 2-ΔΔCt method was used to count the q RT-PCR value and the relative expression level of the target genes was calculated by the values of target genes/ GAPDH. The experiment was repeated three times.

**Flow cytometry**

For evaluation of CD133 and CD26 expression by tumor organoids, organoids were dissociated to single cells using TrypLE Express. For parental biopsies, epithelial tumor cells were isolated by digestion enzyme buffer and FACS-purification (EPCAM+CD45-CD31-FAP-). Cells were washed in FACS buffer (PBS + 5 mM EDTA + 1% bovine serum antigen) and stained with antibodies for 30 min at 4℃. Cells were washed twice with FACS buffer, fixed, and recorded at a Becton Dickinson Fortessa flow cytometer.

**Statistical analysis:**

Student’s *t* test was used to analyze the data. Results are given as mean ± SEM unless otherwise indicated. Probability values < 0.05 were considered significant.

**References:**

1. T. Sato, D.E. Stange, M. Ferrante, et al. Long-term expansion of epithelial organoids from human colon, adenoma, adenocarcinoma, and Barrett's epithelium. *Gastroenterology*. 141;5:1762.

2. A. Calon, E. Espinet, S. Palomo-Ponce, et al. Dependency of colorectal cancer on a TGF-β-driven program in stromal cells for metastasis initiation. *Cancer Cell*. 22;5:571.

3. S.H. Lee, W. Hu, J.T. Matulay, et al. Tumor Evolution and Drug Response in Patient-Derived Organoid Models of Bladder Cancer. *Cell*. 173;2:515.

4. F.A. Ran, P.D. Hsu, J. Wright, et al. Genome engineering using the CRISPR-Cas9 system. *Nat Protoc*. 8;11:2281.

1. T. Sato, D.E. Stange, M. Ferrante, et al. Long-term expansion of epithelial organoids from human colon, adenoma, adenocarcinoma, and Barrett's epithelium. *Gastroenterology*. 141;5:1762.

2. A. Calon, E. Espinet, S. Palomo-Ponce, et al. Dependency of colorectal cancer on a TGF-β-driven program in stromal cells for metastasis initiation. *Cancer Cell*. 22;5:571.

3. S.H. Lee, W. Hu, J.T. Matulay, et al. Tumor Evolution and Drug Response in Patient-Derived Organoid Models of Bladder Cancer. *Cell*. 173;2:515.

4. F.A. Ran, P.D. Hsu, J. Wright, et al. Genome engineering using the CRISPR-Cas9 system. *Nat Protoc*. 8;11:2281.

5. G. Schwank, B.K. Koo, V. Sasselli, et al. Functional repair of CFTR by CRISPR/Cas9 in intestinal stem cell organoids of cystic fibrosis patients. *Cell Stem Cell*. 13;6:653.

**Supplementary figures:**


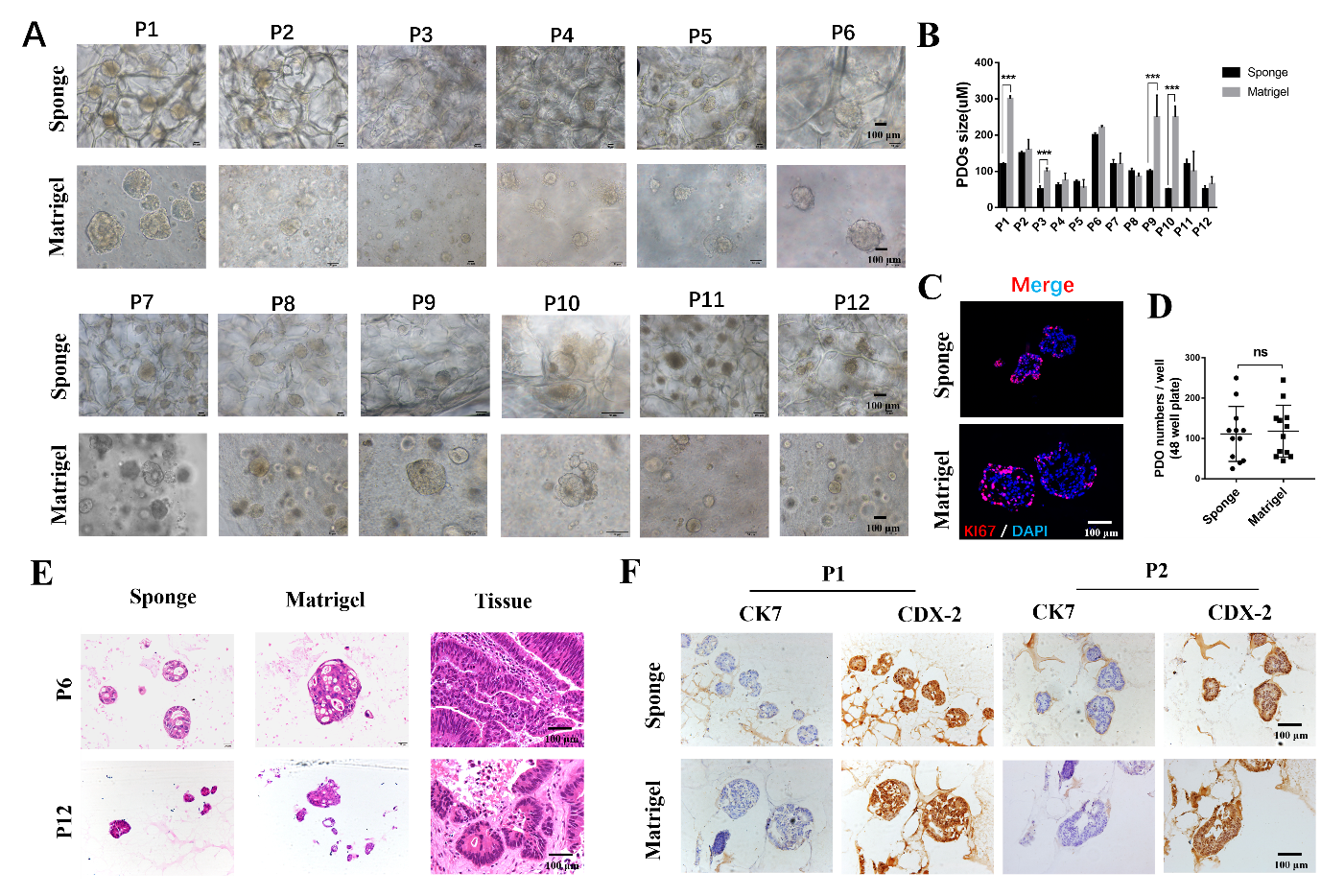


**Figure S1. Overview of PDOs cultures and quantification of the number of mCRC-PDOs formed in HA-Coll sponge or Matrigel.**

**(A)** Freshed isolated tumor cells derived from the same biopsy were seeded into HA-Coll sponges or Matrigel in 48-well plates (5x10^3^/well). **(B and D)** Organoids distribution and diameters were quantified using ImageJ software from phase contrast images of living mCRC-PDOs cultured in HA-Coll sponges or Matrigel on day 14. The spheroids were excluded when the diameters under 10um. This experiment was repeated in all 12 mCRC patients. **(C)** The expression of Ki67 showed the similar proliferation state of PDOs in sponge and Matrigel. **(E)** Representative H&E staining images of PDOs from FO-sensitive SD patients comparing to their parental tumors. **(F)** Representative images of immunohistochemistry staining for the nuclear transcription factor CDX-2 and epithelial cytokeratin CK7. Scale bar, 100μM.

**
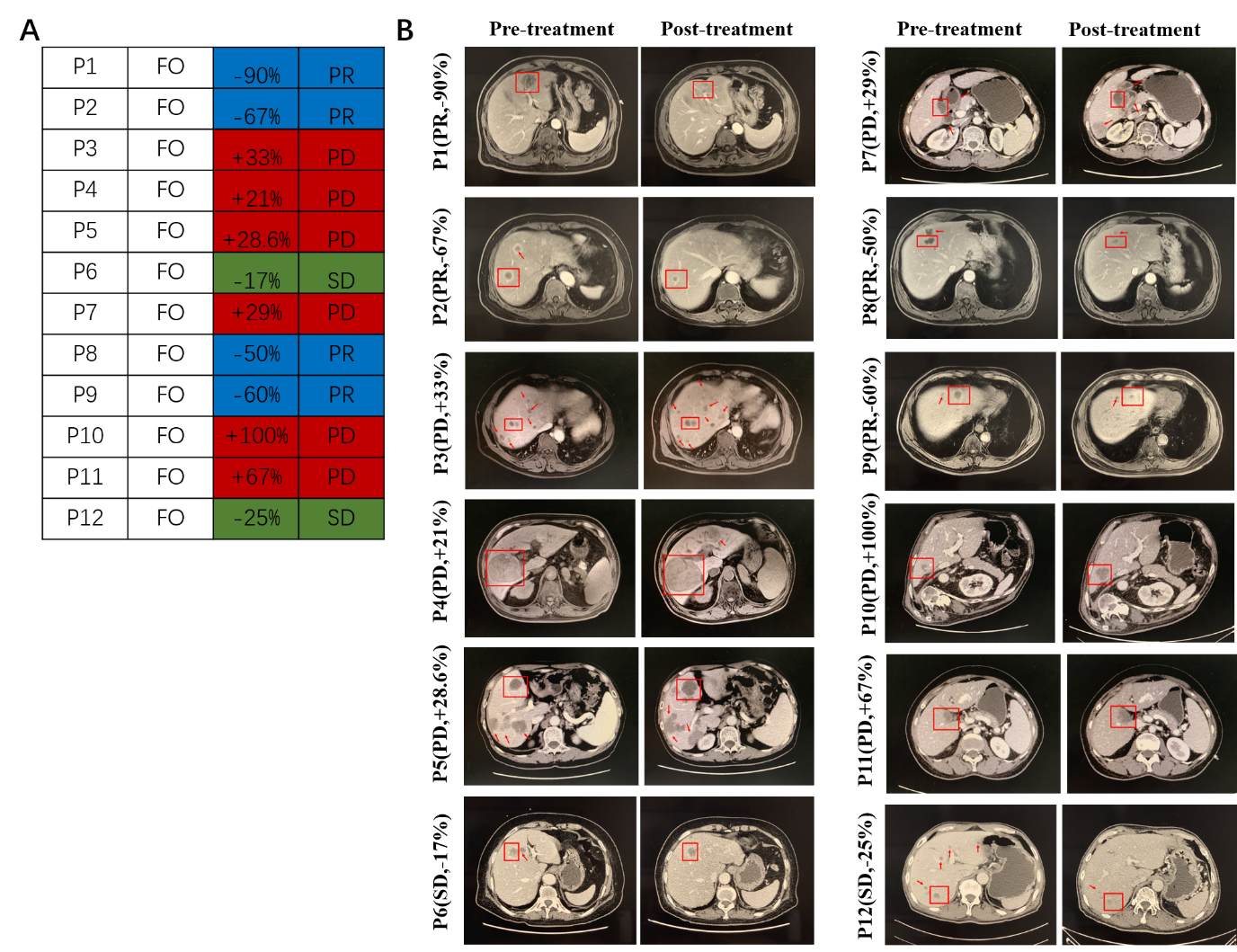
**

**Figure S2. Overview of the FO PDO-patient cohort.**

**(A)** The patient/sample number (p#), treatment (FO = 5-FU plus oxaliplatin), and subsequent response are presented. All patients were biopsied before start of treatment. + or - indicates the percentage of tumor growth or shrinkage compared to the first evaluation (best response). **(B)** CT scans of paired patients before start of treatment and at progression. The text on the left states the patient number, the RECIST response, and the % shrinkage of the target lesion, which is indicated by the red box.

**
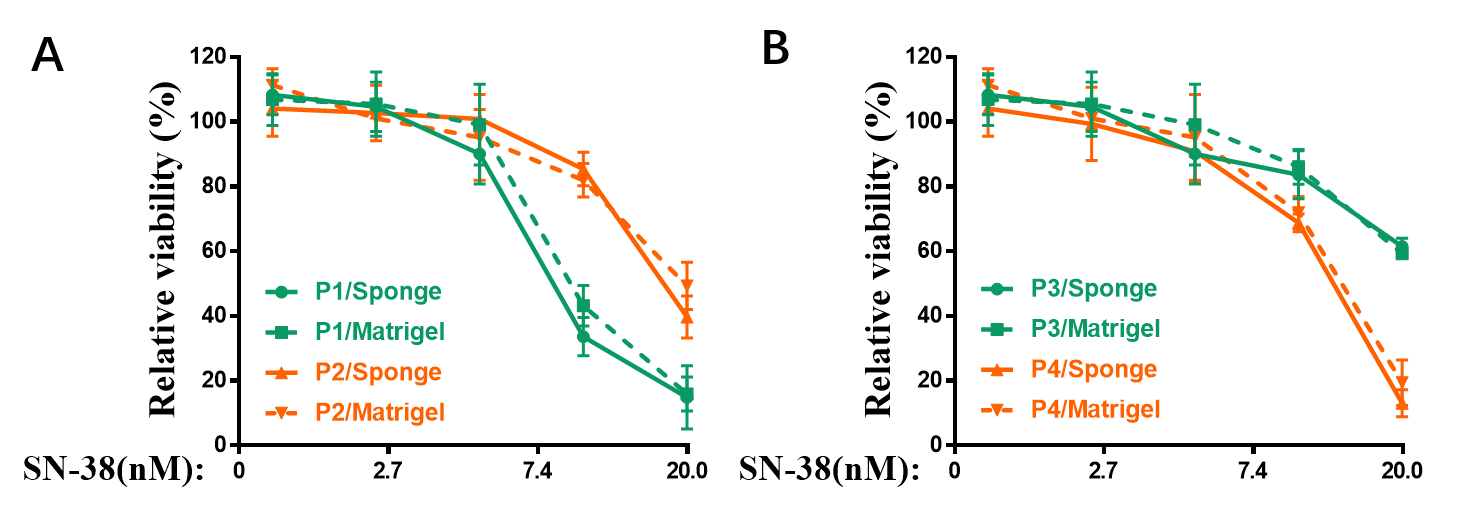
**

**Figure S3:** ***Ex vivo* drug responses with second-line Irinotecan. (A-B)** There were no significant differences of IC50 values between PDOs^Sponge^ and PDOs^Matrigel^ in the representative FO-sensitive patients P1and P2, or FO-resistant P3 and P4.

**
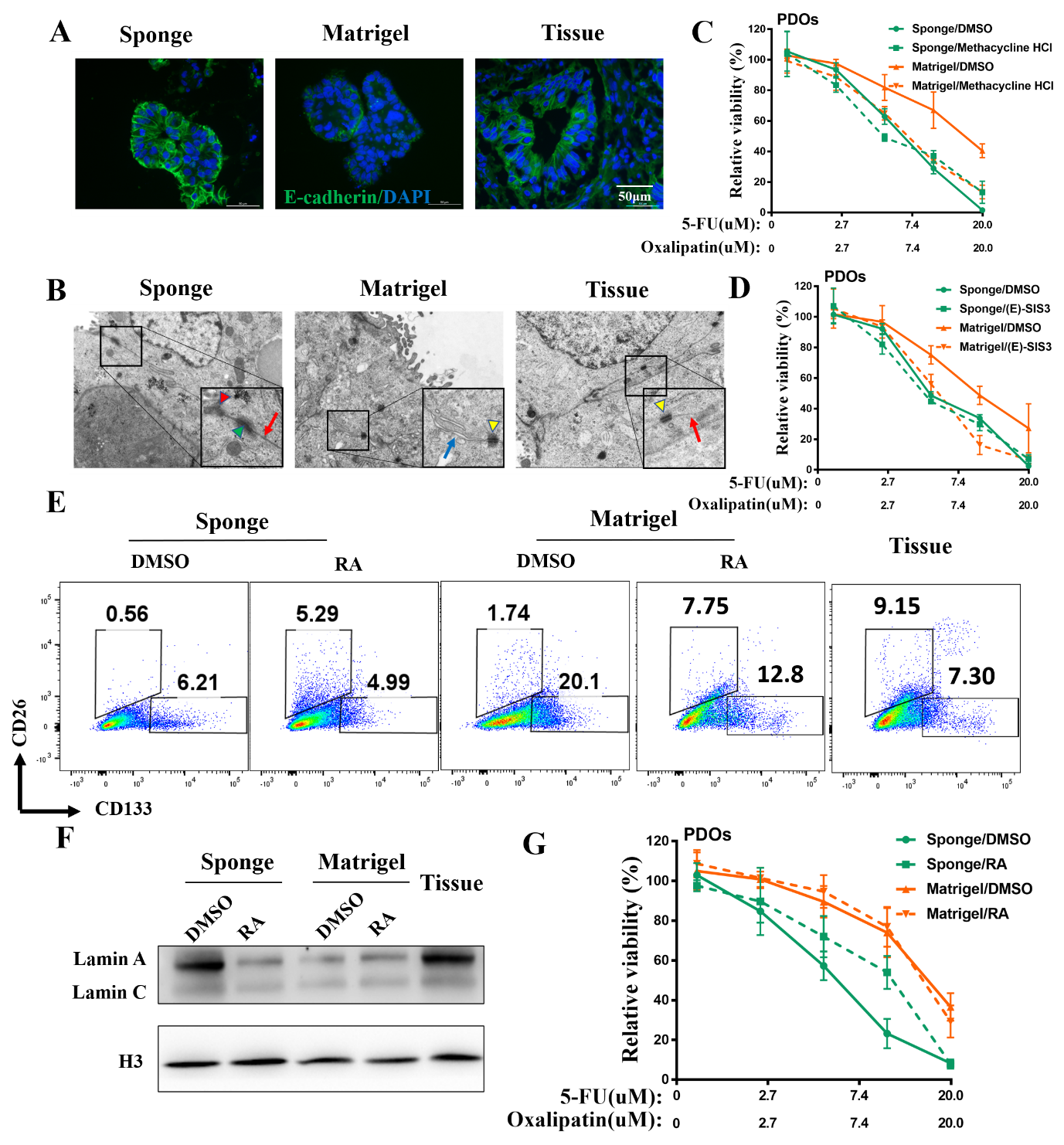
**

**Figure S4. HA-Coll sponge can keep the epithelial and differentiation state** **of PDOs. (A)** The expression levels of E-cadherin in representative PDOs from P2 were examined by immunofluorescence. The epithelial cells of parental biopsy were isolated as control. **(B)** Cell-cell junctions in PDOs^Sponge^ and PDOs^Matrigel^ in representative PDOs from P2 were exaimined by transmission electron microscopy. Red arrowhead for tight junctions; Green arrowhead for adherens junctions; Yellow arrowhead for desmosome; Red arrow for gap junctions; Blue arrow for dissolution of cell–cell junctions. **(C-D)** The *exe vivo* dose-response curves (DRCs) of representative PDOs from P2 exposed to FO regimen after adding the EMT inhibitors: Methacycline HCl or (E)-SIS3. **(E)** Lamin-A contribute to differentiation state of PDOs^sponge^. The proportion of CRC-CSCs in PDOs and parental tissues from P2 were analyzed by flow cytometry. PDOs were pretreated with 5-FU and oxaliplatin for 6 days with or without 1 µM retinoic acid (RA). **(F)** The expression levels of lamin A protein in PDOs and their parental tumor tissues from P2 were examined by immunoblotting. **(G)** The DRCs of PDOs from P2 treated with 5-FU and oxaliplatin in the presence of RA.

**
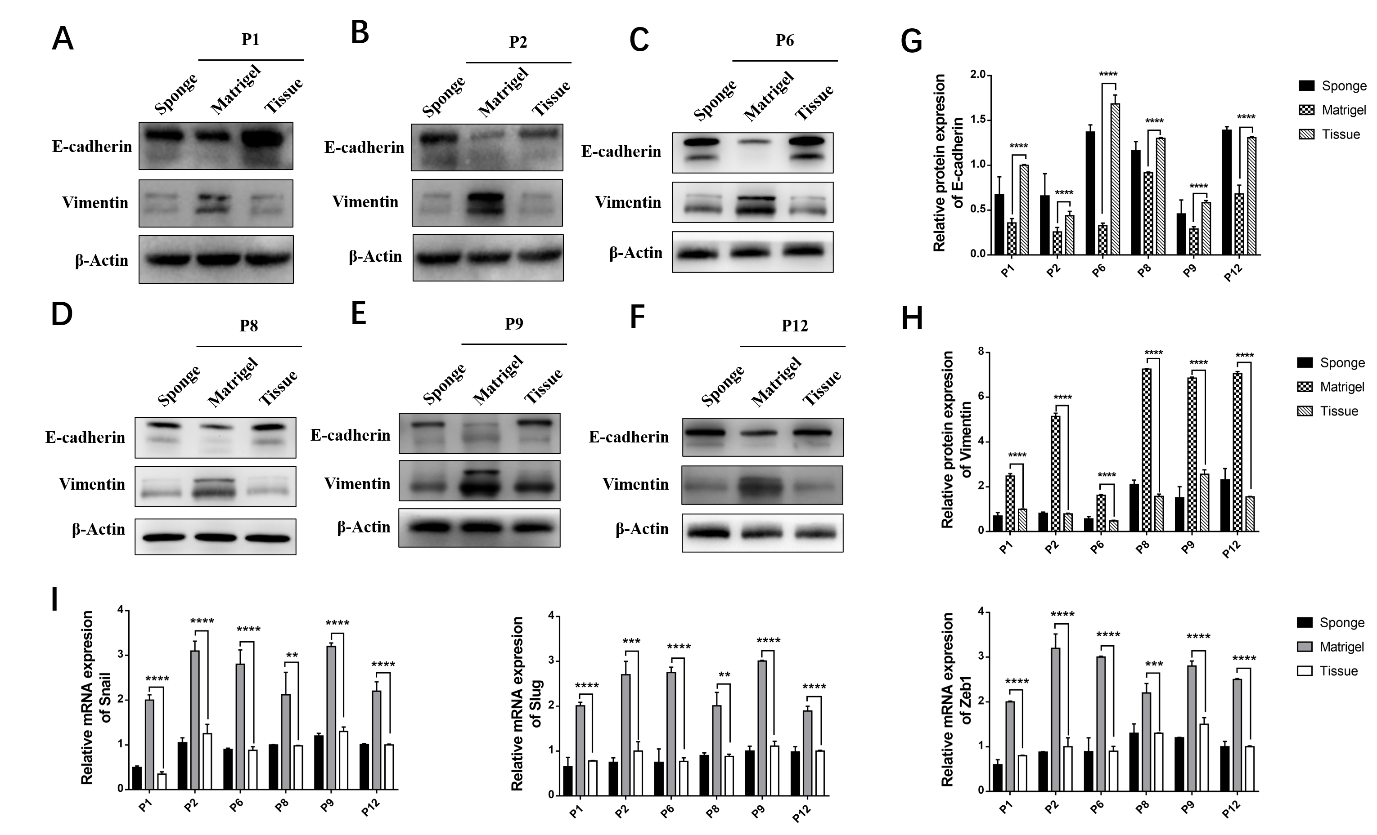
**

**Figure S5. Expression level of EMT-associated proteins in PDOs from PR/SD group patients. (A-F)** Immunoblotting of EMT-associated proteins in PDOs and tissues. **(G-H)** The expression level of E-cadherin and Vimentin from FO-sensitive group was quantified by Image J software. **(I)** The mRNA level of Snail, Slug and Zeb1(EMT markers) by qPCR.


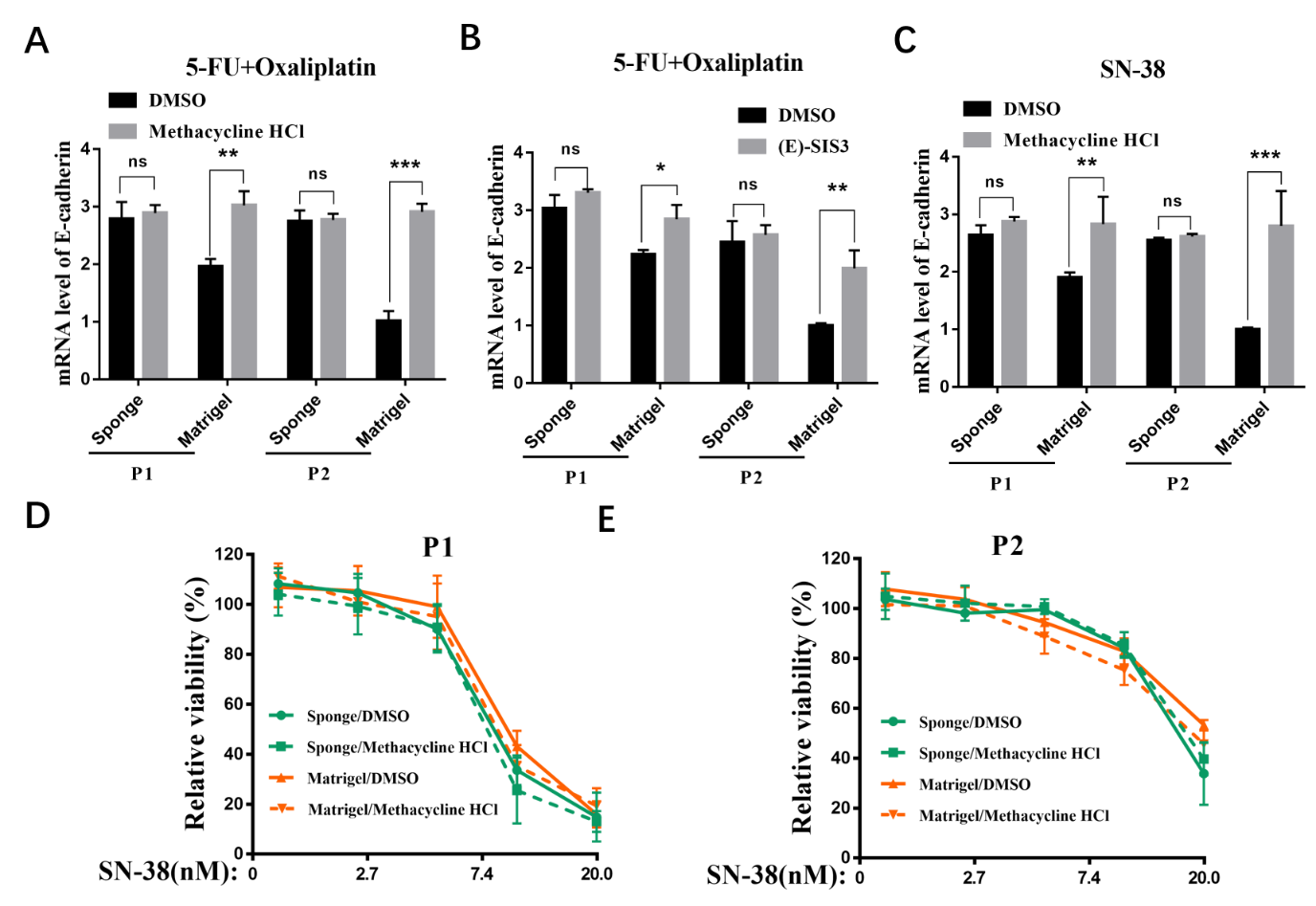


**Figure S6. EMT inhibitors affected the drug-sensitivity of PDOs^Matrigel^ on FO treatment.**

**(A-C)** The effects of EMT inhibitors on the mRNA expression level of E-cadherin in PDOs. The mRNA level of E-cadherin in PDOs was analyzed by qPCR. **(A-B)** The mRNA expression level of E-cadherin in PDOs exposed to FO regimen after adding Methacycline HCl or (E)-SIS3. **(C)** The mRNA expression level of E-cadherin in PDOs exposed to SN-38 in the presence of Methacycline HCl. **(D-E)** The DRCs of PDOs exposed to SN-38 after adding Methacycline HCl**.**

**
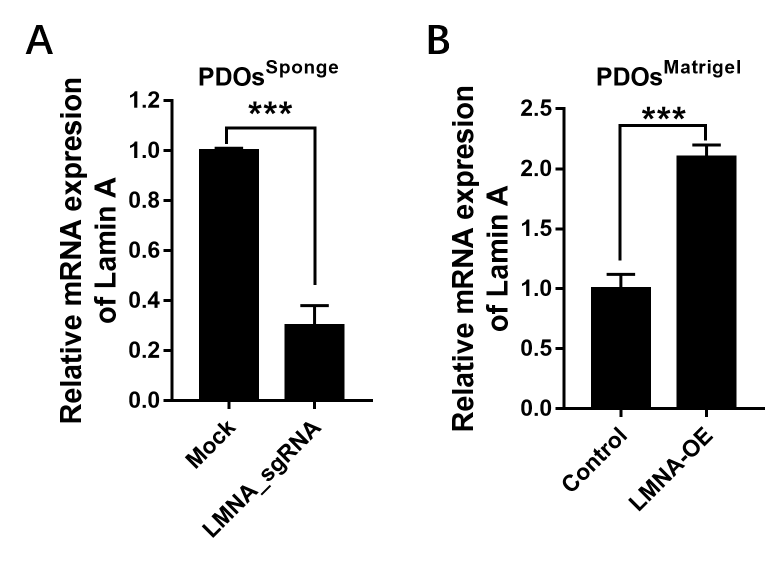
**

**Figure S7**. **Genetic experiments of Knockout / overexpression of LMNA in PDOs^Sponge^ / PDOs^Matrigel^.** **(A)** mRNA levels of Lamin A after *LMNA* KO in PDOs^Sponge^**.** **(B)** mRNA levels of Lamin A after *LMNA* overexpressed in PDOs^Matrigel^.
