# Supplementary material for: Patient‐derived organoids in cellulosic sponge model chemotherapy response of metastatic colorectal cancer
Source: Clin Transl Med. 2021 Jan 12;11(1):e285. doi: 10.1002/ctm2.285 (PMC7803352; doi:10.1002/ctm2.285)
Supplement: Supplementary file 2 — Supporting Information [file CTM2-11-e285-s002.docx]

**Table S1**. Clinical characteristics of patients in our study.

| **No** | **Age** | **Gender** | **Localization** | **MSI status** | **Pathological diagnosis** | **CEA**  **[ng/ml]** | **Biopsied lesion** |
| --- | --- | --- | --- | --- | --- | --- | --- |
| P1 | 63 | Female | Ascending colon | MSS | carcinoma grade Ⅳ | 41.14 | Liver |
| P2 | 66 | Male | Sigmoid colon | MSI | carcinoma grade Ⅳ | 69.4 | Liver |
| P3 | 46 | Female | Rectum | MSS | carcinoma grade Ⅳ | 26.12 | Liver |
| P4 | 67 | Male | Rectum | MSI | carcinoma grade Ⅳ | 7.82 | Liver |
| P5 | 67 | Female | Rectum | MSS | carcinoma grade Ⅳ | 3.44 | Liver |
| P6 | 66 | Male | Descending colon | MSS | carcinoma grade Ⅳ | 17.2 | Liver |
| P7 | 77 | Male | Rectum | MSS | carcinoma grade Ⅳ | 2.45 | Liver |
| P8 | 41 | Female | Right-sided colon | MSS | carcinoma grade Ⅳ | 13.8 | Liver |
| P9 | 65 | Male | Sigmoid colon | MSS | carcinoma grade Ⅳ | 15.7 | Liver |
| P10 | 53 | Male | Rectum | MSS | carcinoma grade Ⅳ | 4.33 | Liver |
| P11 | 56 | Male | Rectum | MSI | carcinoma grade Ⅳ | 12.71 | Liver |
| P12 | 87 | Male | Left-sided colon | MSS | carcinoma grade Ⅳ | 10.77 | Liver |
